# Fusion of Multi‐Paradigm EEG Microstate Features to Enhance the Recognition of Mild Cognitive Impairment

**DOI:** 10.1002/brb3.71398

**Published:** 2026-04-14

**Authors:** Lili He, Xiaolu He, Xiang Li, Shenyi Wang, Chun Ji, Feifei Yin, Xiuchun Yang, Yida He, Pengxiang Zuo, Junfeng Gao

**Affiliations:** ^1^ School of Medicine Shihezi University Shihezi China; ^2^ Department of Biomedical Engineering South‐Central Minzu University Wuhan China; ^3^ Xinjiang Shihezi People's Hospital Shihezi China

**Keywords:** electroencephalography, machine learning (ML), microstate analysis, mild cognitive impairment (MCI), multi‐paradigm

## Abstract

**Background**: Mild cognitive impairment (MCI) represents a transitional stage between normal aging and Alzheimer's disease. Although neuropsychological screening tools such as the Montreal Cognitive Assessment (MoCA) are widely used, they do not directly capture underlying neural dynamics. EEG microstate analysis provides a fast and noninvasive approach to characterize large‐scale brain network activity. However, most previous studies have relied on single‐paradigm recordings. This study proposes a unified framework integrating resting‐state and task‐based EEG microstate features to enhance MCI recognition.

**Methods**: EEG data were acquired from 63 age‐ and sex‐matched participants, comprising 32 patients with MCI and 31 healthy controls, during both resting‐state and Stroop task paradigms. Data augmentation was applied to generate 10 samples per subject, improving model robustness. We extracted 26 microstate features, including global explained variance (GEV), duration, coverage, transition probability, and Lempel–Ziv complexity. Subsequently, statistically informed minimum redundancy maximum relevance (mRMR) selection was used to determine the optimal feature subset (OFS; ≤ 5 features), followed by classification via a support vector machine (SVM) with stratified fivefold cross‐validation.

**Results**: Based on the OFS, resting‐state and task‐based models achieved accuracies of 81.8% and 88.0%, respectively. Multi‐paradigm EEG fusion improved accuracy to 90.3%. MoCA alone achieved 87.6% accuracy. When MoCA was integrated with multi‐paradigm EEG features under the same selection framework, accuracy further increased to 93.8%, with improved balance between sensitivity and specificity.

**Conclusion**: Integrating resting‐state and task‐evoked microstate dynamics enhances MCI classification beyond single‐paradigm EEG. Importantly, EEG features provide complementary diagnostic information beyond conventional cognitive screening, supporting a hybrid electrophysiological–neuropsychological framework for early detection of MCI.

## Graphical abstract

Integrating resting‐state and Stroop task EEG microstate features improves MCI diagnosis, achieving 91.0% accuracy (88.8% sensitivity, 93.1% specificity) and outperforming single‐paradigm analyses (83.1%/87.2%).

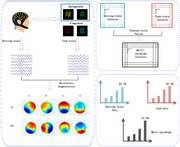

## 1 Introduction

Mild cognitive impairment (MCI) is a clinically defined transitional neurocognitive disorder characterized by a decline in cognitive function greater than expected for normal aging but insufficient to meet diagnostic criteria for dementia (Petersen et al. 1999). It is a common condition in the elderly population and a major clinical concern because of its high conversion rate to Alzheimer's disease (AD), estimated between 40% and 75% (Tahami Monfared et al. 2022). Early identification of MCI is crucial for delaying or preventing the progression to dementia; however, conventional screening tools such as the Mini‐Mental State Examination (MMSE) and Montreal Cognitive Assessment (MoCA) often lack sensitivity for early‐stage detection (Folstein et al. 1975; Nasreddine et al. 2005). These scales inherently rely on patients' behavioral performance and subjective interpretation by assessors, and may be influenced by factors such as educational level, cultural background, and testing state. In contrast, EEG provides a direct and objective neurophysiological measure that captures intrinsic brain dynamics independent of subjects' subjective cooperation or performance. Therefore, EEG‐derived biomarkers hold promise for offering more stable and physiologically grounded objective evidence for the early diagnosis of MCI.

EEG provides a noninvasive, temporally precise window into neural dynamics and has been widely used in the study of neurological disorders, including epilepsy (Hazarika et al. 2025), AD (Gallego‐Viñarás et al. 2025), and Parkinson's disease (Meng et al. 2025). In MCI, EEG biomarkers have been explored for early diagnosis through power spectral density (Mitsukura et al. 2022) and event‐related potential (ERP) analyses (Katayama et al. 2023). Beyond frequency‐domain analysis, EEG microstate analysis captures transient, quasi‐stable scalp potential configurations that reflect large‐scale functional brain network activity (Lehmann et al. 1987). Microstate metrics such as duration, occurrence (OC), coverage (COV), and transition probabilities (TPs) have been shown to exhibit significant alterations in MCI and AD cohorts (Lamoš et al. 2021; Lassi et al. 2023; Z. Li et al. 2024; Musaeus et al. 2019; Tu et al. 2022). Musaeus reported higher OC and COV of microstate Class A in MCI and AD patients (Musaeus et al. 2019), while Lamoš found shorter duration and altered transition dynamics in MCI with Lewy bodies (Lamoš et al. 2021). Tu and Lassi further showed abnormal transitions between symmetric (C, D) and asymmetric (A, B) classes, suggesting disrupted large‐scale neural integration (Lassi et al. 2023; Tu et al. 2022). Li proposed a brain homeostasis coefficient (BhC) to quantify connectivity stability, finding reduced BhC in MCI and AD, particularly in the frontal regions (Z. Li et al. 2024).

Although microstate analysis has demonstrated high sensitivity to MCI‐related neural alterations, several studies have reported substantial inter‐ and intra‐subject variability in commonly used resting‐state EEG network metrics, which may compromise their reliability as robust biomarkers (Van Diessen et al. 2015). In contrast to the resting‐state, which reflects large‐scale intrinsic brain dynamics under minimal cognitive load, task‐based engagement elicits more pronounced and time‐locked cross‐frequency interactions that are central to many cognitive processes. As a result, task‐based EEG provides a more direct window into the electrophysiological mechanisms underlying behavior and cognition (Voytek et al. 2013). Recent evidence further indicates that models predicting individual cognitive performance or pathological phenotypes often achieve superior performance when trained on task‐state data, particularly when the prediction target is linked to specific cognitive functions; in some cases, combining resting‐ and task‐state data can yield complementary benefits (Kaushik et al. 2023; Pashkov and Dakhtin 2025). Emphasizing this point, Faiman et al. (2023) caution that univariate resting‐state measures frequently exhibit limited discriminative power as diagnostic tools, underscoring the risks of relying solely on such indices in clinical decision‐making.

In this study, the Stroop task is selected because it engages core executive control processes, including conflict monitoring and inhibitory control, which are supported by the prefrontal and anterior cingulate cortices (Heidlmayr et al. 2020; Kerns et al. 2004). These functions are particularly vulnerable in MCI (Cipriani et al. 2025). By comparing Stroop‐evoked microstate dynamics with resting‐state patterns, the study aims to capture both baseline and cognitively demanding neural states, thereby increasing sensitivity to subtle disease‐related alterations.

This limitation restricts the ability to capture brain dynamics during active cognitive engagement. Recent multimodal and multi‐paradigm approaches have achieved improved classification accuracy in psychiatric and cognitive disorders by integrating EEG data from different experimental conditions (Cai et al. 2020; Chai et al. 2023; Yang et al. 2023). Building upon this evidence, the present study introduces a unified framework that integrates resting‐state and Stroop task‐evoked EEG microstate features within a support vector machine (SVM) classifier. By combining intrinsic and task‐related brain dynamics, this approach aims to capture complementary neural signatures associated with early cognitive decline and improve classification performance relative to single‐paradigm models. Importantly, to establish clinical relevance, we directly benchmark the proposed EEG framework against the MoCA and evaluate whether EEG microstate features provide incremental diagnostic value beyond conventional cognitive screening.

## 2 Materials

### 2.1 Participants

Participants are recruited from the Department of Neurology at a tertiary hospital in Shihezi City, Xinjiang, China. Diagnoses are made by board‐certified neurologists in accordance with the National Institute on Aging—Alzheimer's Association (NIA—AA) Working Group criteria (Albert et al. 2011). Eligible participants are identified through systematic screening of electronic medical records and must meet the following inclusion criteria: (i) age ≥ 45 years; (ii) subjective memory complaints reported by the patient or an informant; (iii) MoCA score < 26; and (iv) MMSE score ≥ 24. Although MCI is more common in older adults, epidemiological evidence indicates that its prevalence is substantial in adults aged 50 years and older (Bai et al. 2022), and midlife vascular risk factors measured as early as age 45–54 significantly predict late‐life cognitive decline (Wang et al. 2025). Accordingly, an inclusion age of ≥ 45 years was adopted to capture early‐stage MCI cases and identify individuals during this critical preventive window. All diagnoses are independently verified by two senior neurologists (associate chief physicians or above). The cohort consists of 32 patients with MCI (20 males/12 females; mean age = 57.34 ± 4.28 years; MoCA = 19.16 ± 1.48) and 31 healthy controls (HC: 20 males/11 females; mean age = 56.74 ± 3.86 years; MoCA = 25.48 ± 1.48). Groups are matched for age, sex, and education level. Exclusion criteria include: (i) psychiatric disorders; (ii) comorbidities potentially affecting brain function (e.g., major depressive disorder, cerebrovascular events, traumatic brain injury, epilepsy); (iii) history of substance or alcohol abuse; and (iv) current use of psychotropic medications.

### 2.2 EEG Data Acquisition

EEG data were recorded using a 64‐channel actiCHamp amplifier (Brain Products GmbH, Germany) following the international 10–10 system, with a sampling rate of 1000 Hz and electrode impedances kept below 10 kΩ. Data were collected in an electromagnetically shielded room and stored in BrainVision‐compatible format for offline analysis.

## 3 Methods

### 3.1 Paradigm and Preprocessing

During resting‐state recording, participants are instructed to remain relaxed, avoid specific cognitive tasks, and keep their eyes closed while 6 min of EEG are recorded. EEG preprocessing is performed in EEGLAB following standardized procedures (Delorme and Makeig 2004). Raw signals are bandpass‐filtered between 2 and 20 Hz, down sampled to 250 Hz, and re‐referenced to the common average reference (CAR) (Michel and Koenig 2018). The 2–20 Hz bandpass filter was selected to exclude delta (< 2 Hz) and beta/gamma (> 20 Hz) activity. Delta activity is contaminated by slow eye movements and electrode drift, which can artificially prolong microstate durations (MSDs); conversely, beta/gamma activity is susceptible to muscle artifacts and exhibits lower topographic stability. This frequency range optimally captures theta and alpha oscillations, which are central to cognitive processes impaired in MCI, while minimizing artifact contamination and remaining consistent with prior microstate studies in MCI cohorts (Lassi et al. 2023; Musaeus et al. 2019). Epochs exceeding ± 100 µV are removed to minimize ocular and muscular artifacts. Bad channels are automatically detected using variance‐based criteria and interpolated with spherical splines (Perrin et al. 1989). Artifact Subspace Reconstruction (ASR) is applied to suppress transient or high‐amplitude artifacts, and Independent Component Analysis (ICA) is used to remove components related to eye, muscle, and cardiac activity (Chang et al. 2018). Combining ASR and ICA improves artifact correction (Callan et al. 2024). Finally, the first 4 min of artifact‐free EEG are retained for analysis to ensure quality and stability.

**FIGURE 1:**
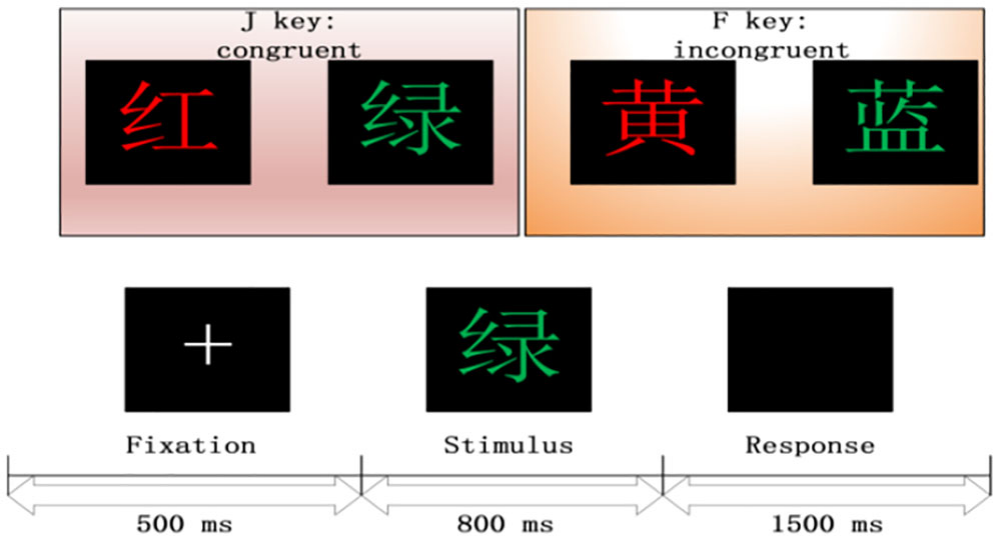
Overview of the Stroop task. Each trial began with a central fixation cross presented for 500 ms, followed by a stimulus displayed for 800 ms. Participants had 1500 ms to respond via a key press. Two conditions were included: (i) congruent, where the word meaning matched its font color (e.g., the word “红”[red] in red), and (ii) incongruent, where the meaning and color were mismatched (e.g., the word “黄” [yellow] in red). The color words used were “红” (red), “黄” (yellow), “蓝” (blue), and “绿”(green).

Participants performed a color–word Stroop task (300 trials, congruent/incongruent 1:1), engaging executive control and conflict monitoring. A schematic diagram of the trial structure is shown in Figure 1. Stroop EEG preprocessing includes electrode localization, bandpass filtering (2–20 Hz), downsampling to 250 Hz, and re‐referencing to CAR. Bad channels are detected and interpolated via spherical splines. Epochs with correct responses to incongruent stimuli are extracted and baseline‐corrected (−200 to 0 ms; analysis window: 0–1000 ms). ICA is used to remove ocular and muscular artifacts. Epochs exceeding ± 100 µV are rejected. Finally, artifact‐free epochs are averaged across trials to improve the signal‐to‐noise ratio (SNR).

### 3.2 Microstate Analysis

The EEGLAB toolbox (Delorme and Makeig 2004) is used to perform cluster analysis on EEG data from both HC and MCI groups to extract microstate templates. Microstate segmentation consists of three main steps:

(i) Detection of global field power (GFP).

Global brain activity is quantified using GFP (Lehmann and Skrandies 1980), defined as:

(1) $$\begin{array}{c} \mathrm{GFP}(t)=\sqrt{\frac{1}{n}\sum_{j=1}^{n}{\left(u_{j}(t)-\bar{u}(t)\right)}^{2}}\; \end{array}$$

where *t* denotes the time point, *u_j_(t)* represents the voltage measured at electrode *j*, and *n* is the total number of electrodes (*n* = 62). $\bar{u}\left(t\right)$ denotes the mean scalp potential across all electrodes at time *t*. GFP reflects the standard deviation of potentials across all electrodes, indicating the overall energy fluctuation of EEG signals. GFP(t) denotes the GFP at each time point t. At GFP peak time points $t_{p}$, we define:

(2) $$\begin{array}{c} \;\mathrm{GF}P_{p}=\mathrm{GFP}(t_{p}) \end{array}$$

where $p=1,\text{…},N_{p}$ indexes the detected GFP peaks. GFP*_p_* represents the discrete sampled values of the continuous function GFP(*t*) evaluated at peak time points. At GFP peaks, the EEG topographic configuration reaches maximal stability. Thus, only data from these peak time points were used for subsequent *k*‐means clustering analysis (Lehmann and Skrandies 1980).

(ii) Microstate clustering analysis

A modified *k*‐means clustering algorithm is applied to GFP‐peak topographies to ensure stable clustering results. The clustering was repeated 20 times for stability (Kim et al. 2021; Tait et al. 2020). Following prior MCI studies, the number of microstate classes was fixed at *k* = 4 (Lassi et al. 2023; Zhao et al. 2022). Let *u*(*t*) ∈ ℝ^n^ denote the vector of scalp potentials at time t, where n is the number of electrodes, and each element $u_{j}\left(t\right)$ corresponds to the voltage at electrode j as defined in Equation (1). Let *C_k_* ∈ ℝ^n^ denote the topographic template (cluster centroid) for microstate *k* (*k* = 1, …, *k*). The *k*‐means objective function is defined as:

(3) $$\min_{\left\{c_{k}\right\}}\sum_{p=1}^{N_{p}}\min_{k}∥u(t_{p})\;-\;C_{k}{∥}^{2}$$

Here, $t_{p}$ denotes the pth GFP peak time point, and $N_{p}$ represents the total number of detected GFP peaks used for clustering. The symbol ||·|| denotes the Euclidean (L2) norm in $R^{n}$. The objective is to determine the set of microstate templates $C_{k}$ that minimizes the sum of squared Euclidean distances between each GFP‐peak topography and its closest microstate template. During the assignment, polarity was ignored by selecting the template with the highest absolute spatial correlation.

(iii) Microstate template fitting

The templates obtained from Step 2 are fitted to the continuous EEG to derive each subject's microstate sequence. In previous studies, smoothing algorithms have been applied to remove microstates shorter than 60–120 ms, which are often considered artifacts (Zhao et al. 2022). However, because neurological dysfunction may shorten MSDs in MCI, smoothing is not applied to preserve potential pathological information (Musaeus et al. 2019).

### 3.3 Feature Extraction

For each subject, the following features are extracted: Global explained variance (GEV), Lempel–Ziv complexity (LZC) (Y. Li et al. 2008), mean duration (MD), COV, OC, and TPs, yielding 26 features in total. GEV quantifies how well the microstate templates explain the EEG signal (Michel and Koenig 2018). Duration, OC, and COV reflect temporal stability and prevalence of each microstate. TPs capture the sequential organization of brain states (Lehmann et al. 2005). LZC indexes the diversity and irregularity of microstate sequences (Y. Li et al. 2008)

GEV is a commonly used metric in EEG microstate analysis (Michel and Koenig 2018), quantifying how well the microstate model explains the EEG signal. It measures the extent to which microstate templates account for the variance in the GFP of the EEG data. GEV is defined as:

(4) $$\begin{array}{c} \mathrm{GEV}=\frac{\sum_{p=1}^{N_{p}}{\mathrm{GFP}}_{p}^{2}\cdot r_{p}^{2}}{\sum_{p=1}^{N_{p}}{\mathrm{GFP}}_{p}^{2}} \end{array}$$

where $N_{p}$ is the total number of detected GFP peaks, $\mathrm{GF}P_{p}$ is the amplitude of the pth GFP peak, and $r_{p}$ is the spatial correlation (Pearson correlation) between the EEG topography at that time point and its corresponding microstate template. Higher GEV values indicate that the microstate templates explain a greater proportion of the EEG signal variance.

The mean MSD refers to the average length of time that a specific microstate class persists continuously in EEG recordings, measured in milliseconds (ms).

The MSD is defined as:

(5) $$\begin{array}{c} \;MD_{k}=\frac{1}{N_{k}}\;\sum_{i\;=1}^{N_{k}}D_{i}^{\left(k\right)} \end{array}$$

where $D_{i}^{\left(k\right)}$ is the duration of the ith instance of microstate k (ms), and $N_{k}$ is the total number of OCs of microstate k. The COV calculates the fractional presence of a microstate class across the complete EEG timespan, computed as (Lehmann et al. 2005; Michel and Koenig 2018):

(6) $$\begin{array}{c} \mathrm{CO}V_{k}=\frac{\sum_{i=1}^{N_{k}}D_{i}}{T} \end{array}$$

where COV*_k_* is the coverage, *N_k_* the occurrence count, and *D_i_* the *i*th duration (ms) of microstate *k*, with *T* being total EEG acquisition time (ms), the OC measures state transition frequency through:

(7) $$\begin{array}{c} OC_{k}=\frac{N_{k}}{T} \end{array}$$

The transition probability, *TP_i,j_* from microstate *i* to *j* is computed as:

(8) $$\begin{array}{c} TP_{i,j}=\frac{N_{i\to j}}{N_{i}} \end{array}$$

where *N_i→j_* is the count of *i* →*j* transitions, and *N_i_* is the total occurrence of microstate *i*. This measure reflects the temporal organization of brain state transitions.

LZC assesses the emergence of new patterns in a symbolic sequence by counting irreducible subsequences (Kaspar and Schuster 1987). The microstate sequences in this study consisted of four distinct classes (A, B, C, D). To avoid overestimating complexity, consecutive identical microstates were merged into a single occurrence. For example, the original sequence “AAAAABBBAAB” was simplified to “ABAB.” The definition of LZC is as follows:

(9) $$\begin{array}{c} \;C_{\left(n\right)}=\sum_{i=1}^{n}u_{i} \end{array}$$

In this formulation, the indicator variable *u_i_* is set to 1 when the extended subsequence *SQ* (i.e., the historical subsequence *S* appended with the new symbol Q) does not exist in the current subsequence set *S*. Otherwise, $u_{i}=0$. Accordingly, the complexity counter *C*_(_ *_n_*_)_ increases by 1 only when *SQ* represents a novel pattern.

To ensure clear identification of feature origins in cross‐paradigm analyses, this study adopted a standardized naming convention. Features extracted from resting‐state and task‐state EEG are denoted by the prefixes “r” and “t,” respectively. Single‐state metrics, such as COV, MD, and OC, are named using the format “paradigm prefix + microstate letter + feature abbreviation.” For instance, tA‐COV represents the COV of Microstate A under task conditions, while rA‐MD denotes the MD of Microstate A during the resting‐state. TPs between microstates are formatted as “paradigm prefix + source state→target state,” such as rC→D, which indicates the TP from Microstate C to D during the resting‐state.

### 3.4 Statistical Analyses

A total of 26 microstate feature metrics were extracted. Group differences were assessed across feature dimensions. Normality was evaluated using the Shapiro–Wilk test, and homogeneity of variance was examined with Levene's test. Features meeting both assumptions were compared using independent‐samples *t*‐tests, whereas features violating either assumption were analyzed with the nonparametric Mann–Whitney *U* test. To control for Type I error inflation from multiple comparisons, all *p*‐values were adjusted using false discovery rate (FDR) correction with the Benjamini–Hochberg procedure.

### 3.5 Machine Learning

To enhance the robustness of machine learning, given the limited sample size, data augmentation was applied to both resting‐state and task‐based EEG. For resting‐state recordings (200 s per subject), continuous EEG was segmented into 10 non‐overlapping 20‐s epochs, providing 10 samples per subject—a duration chosen to balance temporal resolution and microstate statistical reliability. For task‐related EEG, available trials were randomly grouped into 10 blocks and averaged within each block to reduce intra‐subject variability while preserving task‐evoked responses, thus generating 10 stable representative segments per subject. This approach consistently expanded the dataset across paradigms, mitigating overfitting risks while maintaining subject‐level independence.

EEG multimodal fusion was implemented by concatenating resting‐state and task‐based microstate features into a unified feature set. In an additional analysis, MoCA scores were incorporated into the candidate feature space and subjected to the same feature selection and classification framework. Following statistical filtering to exclude non‐significant features, the remaining significant features were directly combined for subsequent machine learning analyses. A wrapper‐based feature selection approach (Sun et al. 2021) was then applied to construct the optimal feature subset (OFS) (Guo et al. 2025). Within this approach, candidate subsets are evaluated using a classifier framework, with performance quantified by accuracy, sensitivity, and specificity. The subset yielding the best performance is retained as the OFS.

SVM classifiers were used as evaluators. Each SVM model was iteratively trained and validated during subset selection to ensure that retained features contributed effectively to classification. Features of each microstate (TFS) were ranked using the minimum redundancy maximum relevance (mRMR) algorithm (Peng et al. 2005). To prevent data leakage, the mRMR ranking and subsequent feature selection were performed exclusively within each training fold during cross‐validation. Ensuring that test‐fold information was not used in any step of feature selection. The open‐source pymrmr package in Python was employed to perform the mRMR ranking (see Supporting Information).

The mRMR algorithm enhances model generalizability by maximizing the relevance (*R*) between features and class labels while minimizing redundancy among features. Mathematically, relevance (*R*) is defined as follows (Peng et al. 2005):

(10) $$\begin{array}{c} R=\frac{1}{\left|S\right|}\;\sum_{x_{i}\in S}I\left(x_{i};C\right) \end{array}$$

Let *S* be the selected features, *C* the class label, and *I*(*xi;C*) their mutual information. Redundancy *D* is:

(11) $$\begin{array}{c} D=\frac{1}{|S{|}^{2}}\;\sum_{x_{i},x_{j}\in S}I\left(x_{i},x_{j}\right) \end{array}$$

*I(x_i_,x_j_)* denotes the mutual information between features *x_i_* and *x_j_*. To optimize feature selection, mRMR employs the following objective function:

(12) $$\begin{array}{c} \max(R-D) \end{array}$$

Features are sequentially added to the subset while evaluating classification accuracy at each step, ultimately determining the OFS.

To evaluate the effectiveness of feature selection, stratified group fivefold cross‐validation (SGKF) was employed. Stratified fivefold cross‐validation with subject‐level partitioning was used to prevent data leakage. To ensure stability, the entire fivefold cross‐validation procedure was repeated five times with different random partitions. The final classification metrics were averaged across all repetitions.

**FIGURE 2:**
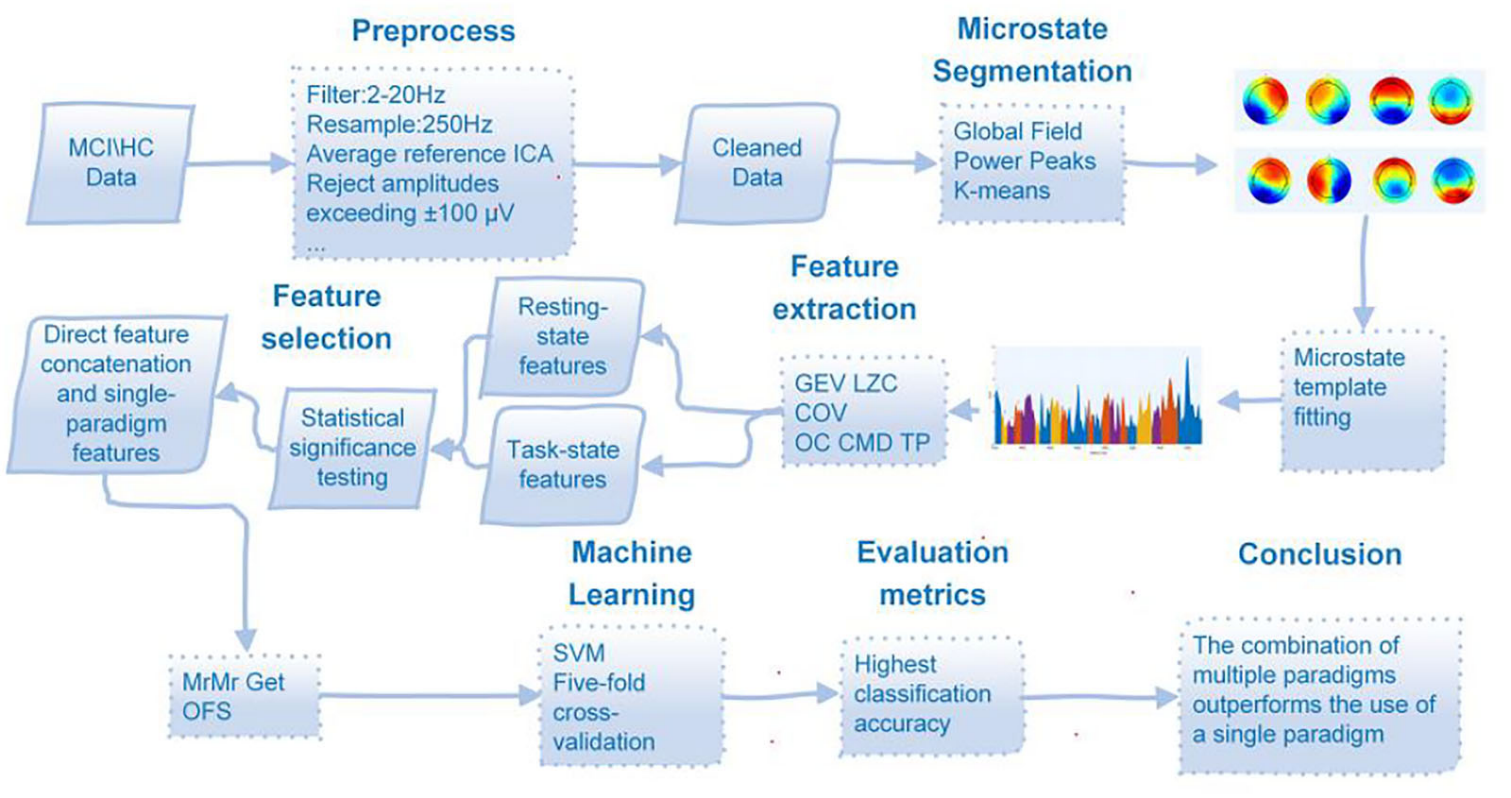
The overall processing workflow.

Within each training fold, hyperparameter optimization for the SVM was conducted using a grid search combined with nested fivefold cross‐validation (Grid Search CV). The SVM was primarily configured with a radial basis function (RBF) kernel, while performance was also compared against a linear kernel to select the best‐performing option. Grid search with nested fivefold validation optimized hyperparameters (C, γ). At each iteration, a nested fivefold validation was used on the training set to identify the optimal hyperparameter combination. The final SVM model, trained with the selected hyperparameters, was subsequently applied to the independent test set for classification. Finally, classification metrics from the five test folds were averaged to provide the overall evaluation criterion. The overall processing workflow is shown in Figure 2.

## 4 Result

### 4.1 Behavioral Results of the Stroop Task

Behavioral performance is summarized in Table 1. Accuracy did not differ between groups. Both congruent and incongruent reaction times were significantly longer in the MCI group compared with the HC group. Group differences were confirmed using both Student's *t*‐test and Welch's *t*‐test, demonstrating the robustness of the results.

**TABLE 1:** Behavioral performance in the Stroop task for HC and MCI groups.

Effect	HC (mean ± SD)	MCI (mean ± SD)	Between‐group *t*‐test	Between‐group Welch's *t*‐test
Accuracy	0.843 ± 0.165	0.824 ± 0.128	*t* = 0.441, *p* = 0.661	*t* = 0.443, *p* = 0.660
Congruent RT (ms)	721.3 ± 121.96	816.05 ± 156.26	*t* = −2.372, *p* = 0.022	*t* = −2.36, *p* = 0.023
Incongruent RT (ms)	814.88 ± 136.24	908.49 ± 138.23	*t* = −2.387, *p* = 0.021	*t* = −2.386, *p* = 0.021

*Notes*: For accuracy, there was no statistically significant difference between HC and MCI groups. For congruent and incongruent RT, there were statistically significant differences between HC and MCI groups. See text for details. *t* represents the *t*‐statistic from the independent samples *t*‐test.

Abbreviations: HC = healthy controls; MCI = mild cognitive impairment; RT = reaction time; SD = standard deviation.

### 4.2 Differences in Microstate Templates

**FIGURE 3:**
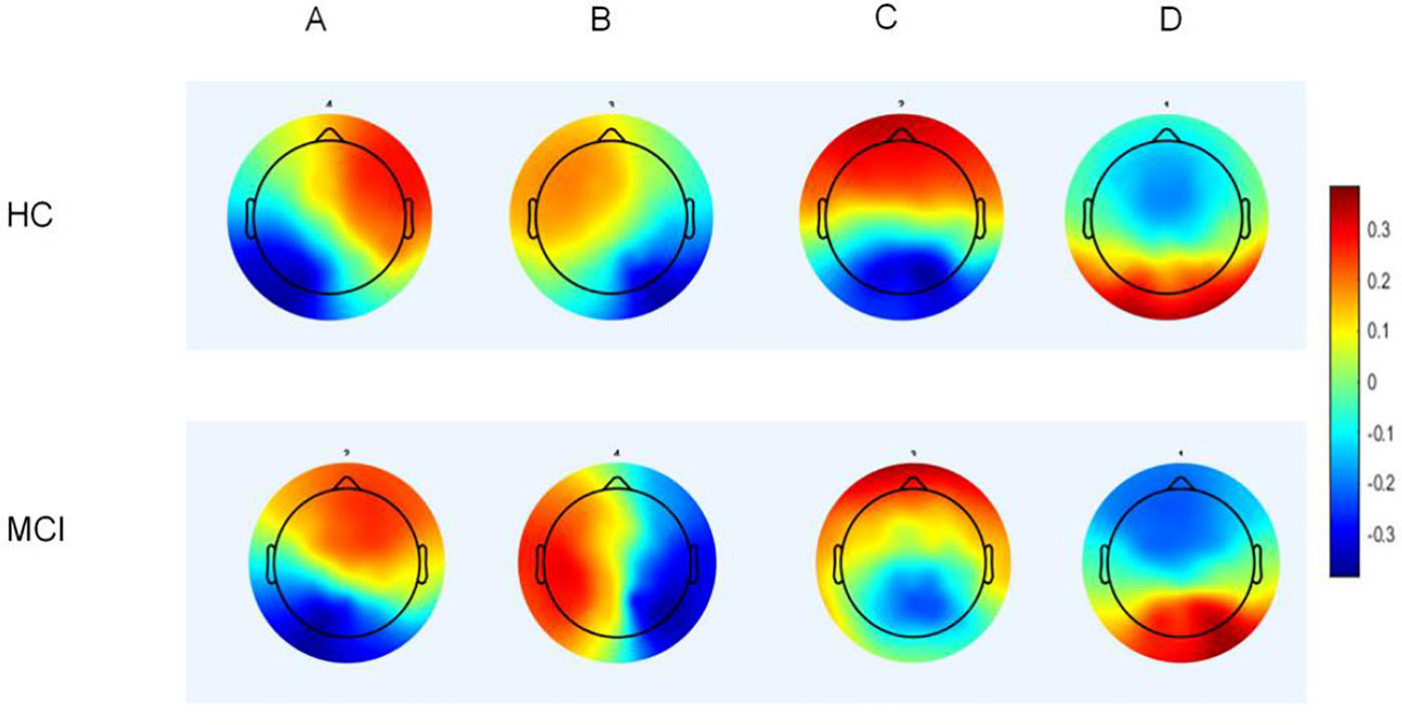
Clustering of HC and MCI groups.

Following microstate clustering, the mean GEV values were 0.755 for the HC group and 0.753 for the MCI group, indicating satisfactory explanatory power in both datasets. The clustering results are presented in the Figure 3. The derived microstate topographies were generally consistent with the canonical four‐class microstate model.

Microstate A exhibited a left anterior–right posterior polarity pattern, typically associated with auditory and language processing networks. Microstate B showed a right anterior–left posterior polarity configuration, commonly linked to visual network activity. Microstate C demonstrated clear frontoposterior antisymmetry, with opposing electrical potentials between anterior and posterior regions. This configuration has been associated with the default mode network (DMN) and internal cognitive processes. Microstate D showed bilateral polarity distributions in the temporoparietal regions. This pattern may correspond to the attentional control network, which is involved in the regulation and executive control of external tasks (Michel and Koenig 2018; Tarailis et al. 2024). Among the four classes, Microstate B exhibited the most pronounced differences between HC and MCI groups, although the topographic configurations remained identifiable.

### 4.3 Between‐Group Statistical Comparisons

**FIGURE 4:**
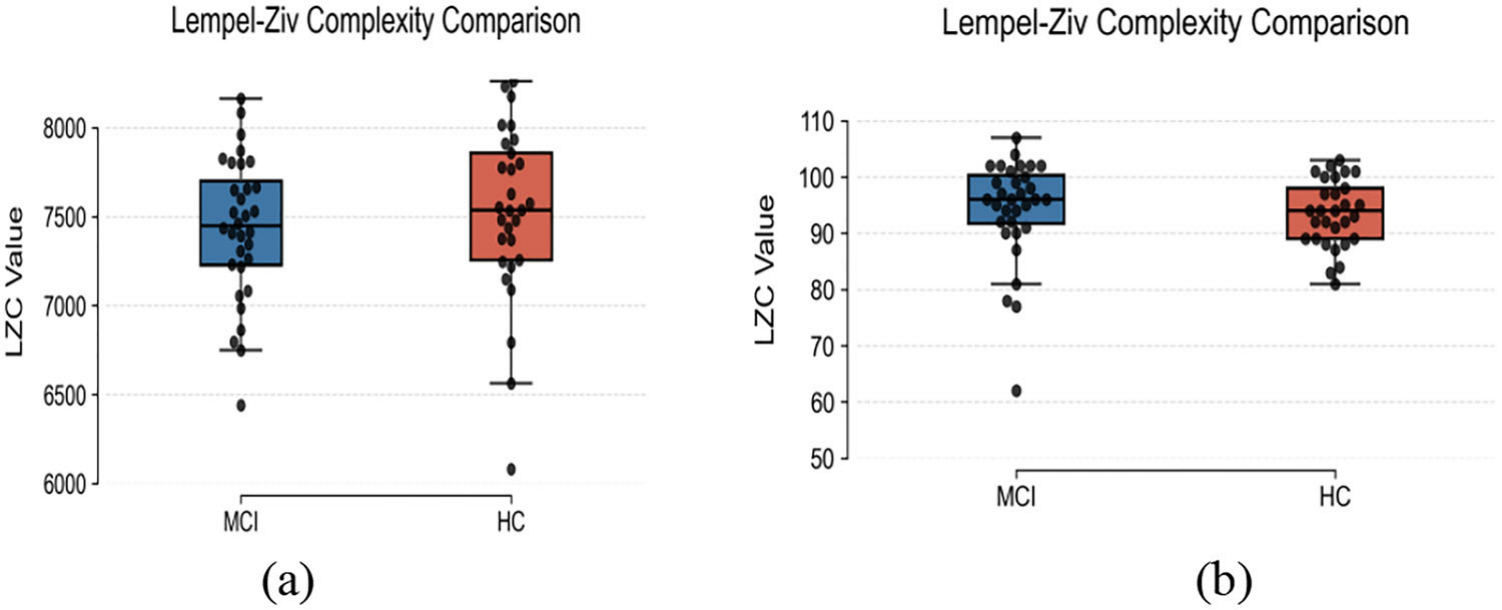
Comparison of Lempel–Ziv complexity (LZC) between MCI and HC under resting‐state (a) and task‐based (b) paradigms. Boxplots with overlaid swarm plots display individual data distributions. No significant group differences were observed in either condition.

Results indicated that GEV and LZC did not differ significantly between resting‐state and task conditions. LZC was employed to quantify the irregularity of brain activity patterns (Figure 4).

**FIGURE 5:**
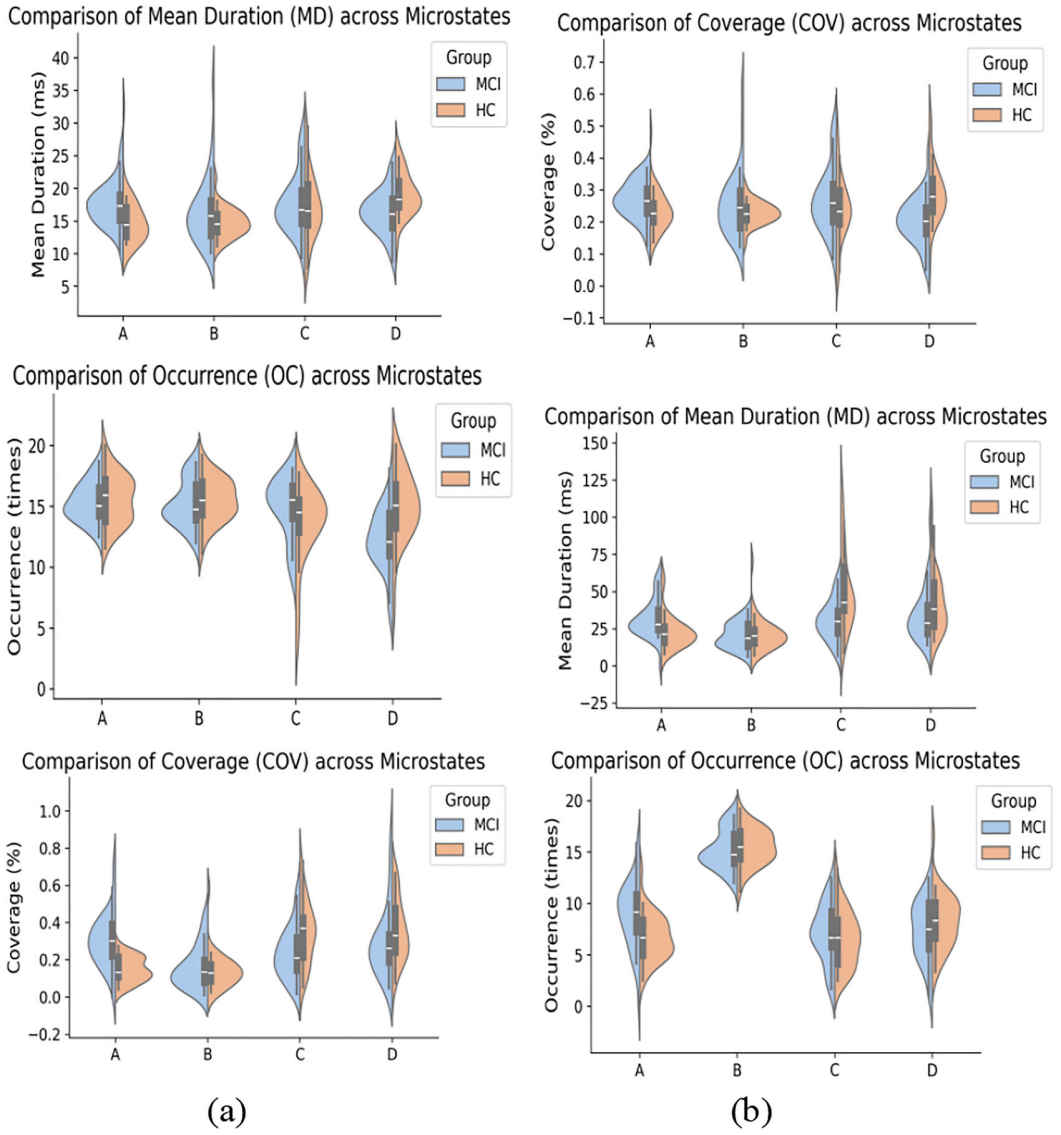
Group differences in microstate dynamics (duration, coverage, and occurrence frequency) between HC and MCI. Violin plots depict data distributions, with embedded boxplots indicating the median and interquartile range. (a) resting‐state EEG; (b) task‐based EEG.

In the resting‐state, MCI patients exhibited a significantly longer MD of Microstate A compared with HCs. In contrast, Microstate D showed a shorter MD, reduced temporal COV, and lower OC frequency (Table 2). During the task state, MCI patients demonstrated a longer MD, greater temporal COV, and higher OC frequency of Microstate A. Conversely, Microstate C exhibited a shorter MD and reduced temporal COV compared with HC (Table 3). The MD, temporal COV, and occurrence frequency of the four microstates are summarized in Figure 5.

**TABLE 2:** Resting‐state microstate feature differences between groups (excluding transitions).

Feature	MCI (mean ± SD)	HC (mean ± SD)	Test method	*p*_FDR
rA‐MD	17.33 ± 3.30	14.43 ± 4.81	Mann–Whitney *U*	0.0055
rD‐MD	16.40 ± 3.40	19.13 ± 3.42	*t*‐test	0.0078
rD‐COV	0.21 ± 0.07	0.29 ± 0.09	*t*‐test	0.0010
rD‐OC	12.24 ± 2.60	14.98 ± 2.96	*t*‐test	0.0013

**TABLE 3:** Task‐state microstate feature differences between groups (excluding transitions).

Feature	MCI (mean ± SD)	HC (mean ± SD)	Test method	*p*_FDR
tC‐MD	29.96 ± 13.47	42.86 ± 27.80	Mann–Whitney *U*	0.0023
tA‐MD	27.83 ± 28.54	21.45 ± 11.75	Mann–Whitney *U*	0.0018
tC‐COV	0.24 ± 0.14	0.35 ± 0.17	*t*‐test	0.0206
tA‐COV	0.30 ± 0.14	0.13 ± 0.07	Mann–Whitney *U*	< 0.0001
tA‐OC	8.91 ± 3.28	6.72 ± 2.26	*t*‐test	0.0105

**FIGURE 6:**
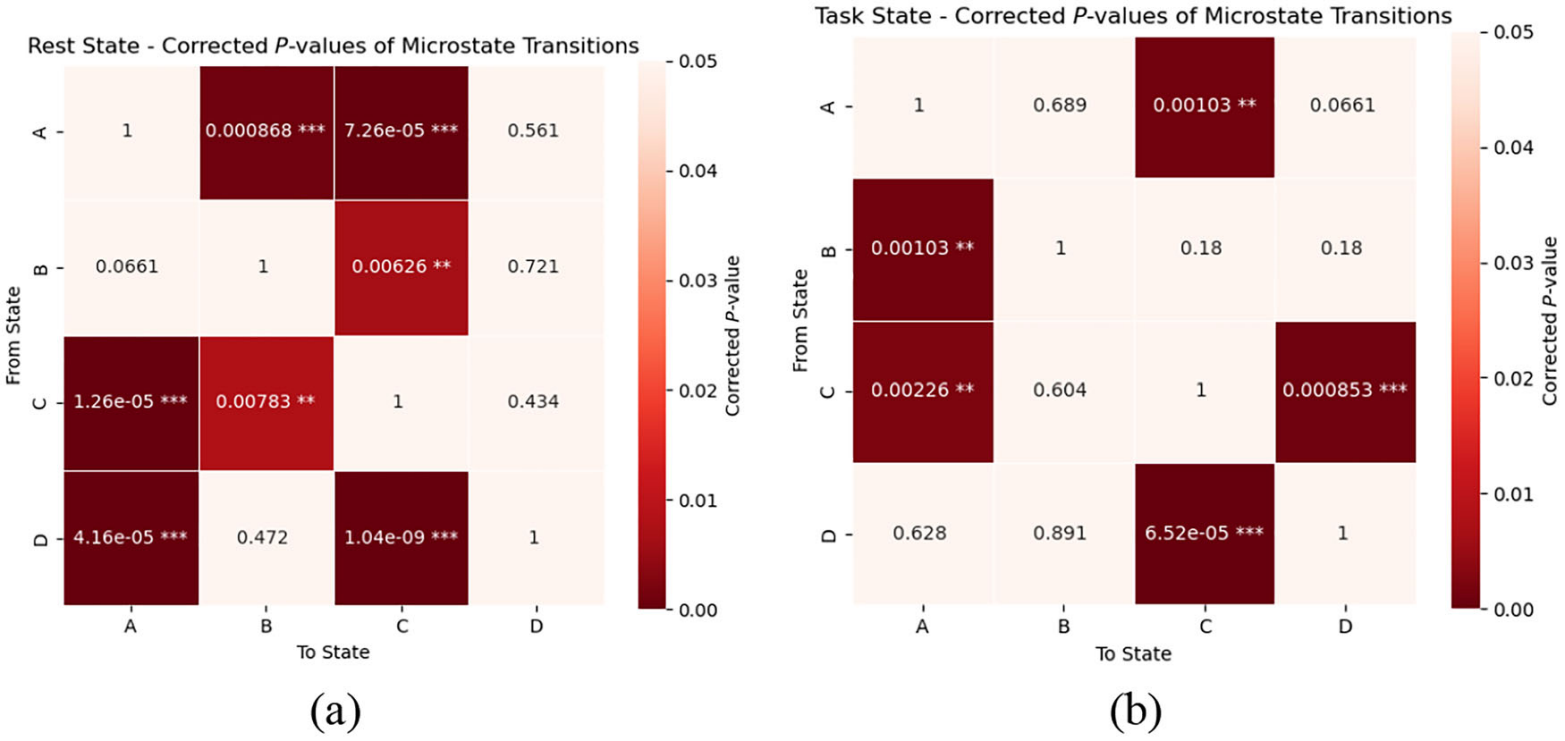
Microstate transition probability heat maps in resting‐state and task‐state conditions. The matrix represents the statistical significance (*p*‐values) of group differences (HC vs. MCI) in transition probabilities between microstates A–D. Warmer colors (dark red) indicate lower *p*‐values. Each cell is annotated with the corresponding *p*‐value and significance level (*p < 0.05, **p < 0.01, ***p < 0.001). (a) Resting‐state EEG, (b) task‐based EEG.

Further analysis revealed significant group differences in TP between microstates, with corresponding heat maps presented in Figure 6 for both conditions.

### 4.4 Machine Learning Classification Results

To balance classification performance with feature space complexity, we first applied statistical significance filtering, followed by mRMR ranking and cross‐validation, constraining the dimensionality of the OFS to a maximum of five features. After statistical analysis, 10 features remained for the task condition, and 13 features remained for the resting‐state condition. Consequently, when combining both conditions, the fused condition contained a total of 23 features. For each feature Count F, the subset yielding the highest classification accuracy among all candidates was selected as the OFS. This strategy reduced feature redundancy while retaining interpretability.

**FIGURE 7:**
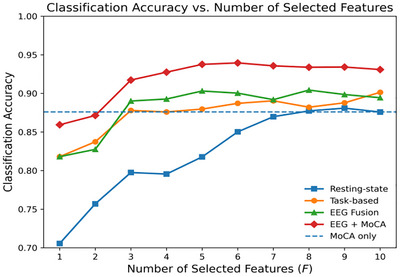
Classification accuracy versus the number of selected features (*F* = 1–10) under resting‐state, task‐based, multimodal fusion, and EEG + MoCA conditions. The dashed line indicates the classification accuracy achieved by MoCA alone (87.6%).

As shown in Figure 7, MoCA alone achieved a classification accuracy of 87.6% under the same validation framework. For EEG‐based models, the highest task‐state classification accuracy (88.0%) was achieved with the OFS comprising tC‐MD, tA‐COV, tC→D, tD→C, and tB→A. In the resting‐state, an accuracy of 81.8% was obtained using rC‐OC, rD‐COV, rD→A, rC→A, and rA→B. Notably, under the multi‐paradigm fusion strategy, the OFS consisting of five EEG features (tD→C, tC→D, rD‐COV, rA→B, and tA‐COV) achieved the highest accuracy of 90.3%. When MoCA was incorporated into the candidate feature pool and subjected to the same feature selection procedure, the resulting OFS (comprising MoCA, tD→C, tC→D, rD‐COV, and tA‐COV) achieved the highest overall classification accuracy of 93.8%.

As summarized in Table 4, the combination of statistical filtering and mRMR effectively reduced dimensionality while producing compact, highly discriminative feature subsets. Importantly, the integration of MoCA within the same optimization framework further improved performance, suggesting complementary contributions from behavioral screening and neurophysiological microstate dynamics.

**TABLE 4:** Classification performance based on the optimal feature subset (OFS).

Type	Accuracy (mean ± SD)	Sensitivity (mean ± SD)	Specificity (mean ± SD)
Resting‐state	81.8 ± 7.8%	81.2 ± 12.0%	82.6 ± 9.7%
Task‐based	88.0 ± 3.8%	85.9 ± 7.0%	90.1 ± 6.7%
EEG multimodal fusion	90.3 ± 4.0%	89.2 ± 8.7%	91.5 ± 4.2%
MoCA only	87.6 ± 8.6%	81.9 ± 14.4%	93.3 ± 14.4%
EEG + MoCA	93.8 ± 3.1%	93.2 ± 4.5%	94.4 ± 6.5%

## 5 Discussion

### 5.1 Resting‐State Microstate Alterations

The microstate topographies closely resembled the canonical four‐class configuration, indicating overall preservation of spatial patterns across groups. Significant temporal alterations were found: Microstate A showed prolonged duration, whereas Microstate D exhibited reduced duration, COV, and OC, reflecting disrupted baseline neural dynamics in MCI. Prolongation of Microstate A likely reflects aberrant engagement of internal cognitive processes such as language processing and DMN activity (Zhou et al. 2023). The research team led by Xiaotian Wu found MCI displayed altered microstate dynamics, with significantly longer COV and duration in Microstate C but shorter in Microstates A, B, and D compared to HCs (Wu et al. 2025). Conversely, Microstate D—typically associated with the salience network (SN) and affective processing—displayed marked reductions in duration, COV, and OC, suggesting impaired salience detection, emotional regulation, and self‐referential processing (Lian et al. 2021). Such abnormalities may underlie clinical symptoms of MCI, including emotional blunting and reduced social engagement. Taken together, these findings indicate a functional imbalance in MCI characterized by hyperactive internal cognitive processes and diminished capacity for external stimulus processing.

### 5.2 Task‐Related Microstate Alterations and Behavioral Correlates

During the Stroop task, MCI patients exhibited significantly increased MD, temporal COV, and OC of Microstate A, suggesting enhanced reliance on the neural processes indexed by this state. These alterations may represent compensatory recruitment of additional neural resources, although prolonged Microstate A duration indicates inefficient processing. Conversely, Microstate C showed reduced duration and COV, consistent with diminished engagement of networks supporting attentional control, cognitive monitoring, and working memory (Lian et al. 2021). These electrophysiological alterations were mirrored in behavioral performance: although accuracy did not differ between groups, MCI patients exhibited significantly prolonged reaction times, indicating a compensatory but less efficient processing strategy. Combined with resting‐state abnormalities, these results highlight Microstate A as a potential electrophysiological biomarker for early MCI detection.

### 5.3 Complexity Measures

LZC quantifies the global irregularity of microstate sequences, reflecting the diversity of spatiotemporal transitions. Our findings indicate that early neural dysfunction in MCI is characterized primarily by local alterations—such as changes in MSD or transition structure—rather than by substantial reductions in overall sequence complexity. Previous evidence suggests that LZC is more sensitive to higher‐order, non‐Markovian dynamics and exhibits pronounced changes in AD (Tait et al. 2020). However, in MCI, the fundamental dynamic architecture may remain relatively preserved, making group‐level differences less detectable. Lassi reported reduced LZC in MCI relative to subjective cognitive decline (SCD), which at first appears inconsistent with our results (Lassi et al. 2023). This discrepancy likely reflects stage‐dependent sensitivity: LZC may capture subtle disruptions during preclinical stages (SCD), but exhibit diminished discriminative power once pathology progresses, where variability across individuals becomes more pronounced. Methodological factors may also contribute. To preserve transient pathological states, smoothing was avoided, potentially increasing variability. In contrast, prior work applying stronger signal regularization may have enhanced the detectability of small effects. Together, these findings suggest that LZC may be more suitable for detecting early trends in cognitive decline rather than differentiating discrete disease stages.

### 5.4 Integration of Resting‐State and Task‐State Mechanisms

Resting‐state microstates reflect intrinsic activity patterns linked to cognitive status (Van De Ville et al. 2010). In contrast, task‐state features capture dynamic, region‐specific network reconfigurations during cognitive load. The most discriminative task‐state features (tC→D, tD→C) primarily reflect directional changes in inter‐regional information flow, consistent with the “task‐induced network reconfiguration” hypothesis (Shine et al. 2019). This pattern suggests that MCI patients engage additional compensatory connections to sustain task performance. Resting‐state features (e.g., rD→C, rA→B) predominantly capture aberrant intrinsic connectivity within the DMN and SN—two hubs consistently implicated as biomarkers of early cognitive decline (Chow et al. 2022; Tarailis et al. 2024). Notably, the retained resting‐ and task‐state features indicate bidirectional modulation of information flow across states (e.g., resting‐state rA→B vs. task‐state tB→A), suggesting that baseline integration inefficiencies in MCI are exacerbated under task demands. This observation aligns with Shine's “dual‐state decoupling model,” which proposes that disease‐related network disruptions not only persist during rest but are dynamically amplified under cognitive stress (Peng et al. 2005).

The Stroop task engages frontoparietal control networks to resolve color–word conflict (Fan et al. 2025). Behavioral evidence shows that while accuracy in MCI remains comparable to controls, reaction times are significantly prolonged, indicating preserved correctness at the expense of efficiency. This aligns with electrophysiological findings: the increased duration and coverage of Microstate A during task execution reflect compensatory over‐engagement of internal cognitive and language‐related networks. Furthermore, enhanced top‐down signaling (tB→A) and attenuated baseline connectivity (rA→B) suggest a trade‐off between task‐state resource allocation and resting‐state resilience. These results support a compensatory but inefficient neural strategy underlying preserved accuracy but prolonged reaction time in MCI (Harrison et al. 2011).

### 5.5 Classification Performance

Task‐based microstate features achieved higher classification accuracy than resting‐state features, suggesting that task‐evoked EEG dynamics may be more sensitive to early cognitive impairment. During cognitive engagement, neural systems are required to flexibly reconfigure in response to increased demands, and such adaptive reorganization may expose subtle dysfunction that remains less evident under resting conditions. In contrast, resting‐state features primarily reflect stable intrinsic connectivity patterns and may overlook transient compensatory processes that emerge during task performance. As a clinical benchmark, MoCA alone demonstrated robust classification performance, consistent with prior validation studies (Ciesielska et al. 2016). Importantly, integrating EEG microstate features with MoCA scores within the same feature‐selection framework further improved classification accuracy beyond both multimodal EEG fusion and MoCA alone. This synergistic gain suggests that behavioral screening and electrophysiological dynamics capture complementary aspects of MCI‐related alterations. While microstate dynamics index real‐time neural coordination across resting and task conditions, MoCA performance likely reflects global cognitive reserve and compensatory capacity that may not be fully expressed during brief EEG recordings. The differential contributions of resting‐state and task‐based features further support this complementarity. Resting‐state measures characterize relatively stable, trait‐like connectivity patterns, whereas task‐based measures capture adaptive reconfiguration under cognitive load. Their integration enhances discriminative power by combining intrinsic network organization with context‐dependent functional responsiveness, thereby capturing both intrinsic and state‐dependent neural signatures of MCI and offering a more comprehensive characterization of disease‐related alterations (Liu et al. 2024; Tu et al. 2022). Moreover, the retained cross‐paradigm features (e.g., rA→B, tB→A) highlight that interactions between states—rather than within‐state metrics alone—may hold the greatest diagnostic relevance. These results extend prior work showing altered microstate transition dynamics in MCI and AD (Lian et al. 2021), and align with studies demonstrating improved classification performance through multimodal microstate feature integration (Wu et al. 2025; Shi et al. 2022). Collectively, the findings emphasize the methodological and clinical advantages of multi‐paradigm fusion, providing a robust framework for early detection and mechanistic understanding of state‐dependent neural dysfunction in MCI.

## 6 Conclusion

Our findings confirm that a combined resting‐state and task‐state EEG microstate framework significantly improves MCI classification performance compared to single‐paradigm analyses. Dynamic microstate patterns, especially TPs and temporal persistence metrics, were identified as particularly discriminative features for early detection. Although the primary feature ranking was performed within nested cross‐validation, the initial statistical filtering step was conducted on the full dataset, which may introduce a slight optimistic bias. Future studies should adopt fully nested preprocessing pipelines. Nevertheless, the relatively small participant pool (*N* = 63, augmented to 630 samples for machine learning) remains a limitation. While data augmentation improved model robustness within this dataset, it cannot fully replace a larger and more diverse cohort. Therefore, the limited number of original participants (*N* = 63) remains a constraint on the external validity of our findings. Future research should seek to validate the proposed EEG microstate‐based biomarkers in larger, multi‐center cohorts encompassing broader demographic and cognitive variability. Such efforts would help establish the robustness and clinical utility of the approach for early MCI detection.

## Author Contributions

Study concept and design: Lili He and Xiaolu He. Data acquisition: Lili He, Feifei Yin, and Xiuchun Yang. Data analysis and interpretation: Xiaolu He, Xiang Li, and Yida He. Drafting of the manuscript: Xiaolu He and Lili He. Statistical analysis: Shenyi Wang and Chun Ji. Study supervision: Pengxiang Zuo and Junfeng Gao. All authors have approved the final version of the manuscript before submission.

## Funding

This study was supported by the Xinjiang Production and Construction Corps' Scinence and Technology Project (Grant Number: 2023AB048, 2023ZD033), and the Fundamental Research Funds for the Central Universities of South‐Central Minzu University (Grant Number: CZZ24015, CZZ25009)

## Ethics Statement

This study strictly adhered to the Declaration of Helsinki principles. Written informed consent was obtained from all participants prior to enrollment. The experimental protocol received formal approval from the Ethics Committee of the First Affiliated Hospital of Shihezi University (Approval No.: KJ2024‐508‐02).

## Conflicts of Interest

The authors declare no conflicts of interest.

## Supporting information




**Supplementary Materials**: brb371398‐sup‐0001‐SuppMat.docx

## Data Availability

Data will be made available on request.
