# Supplementary material for: Fusion of Multi‐Paradigm EEG Microstate Features to Enhance the Recognition of Mild Cognitive Impairment
Source: Brain Behav. 2026 Apr 14;16(4):e71398. doi: 10.1002/brb3.71398 (PMC13080112; doi:10.1002/brb3.71398)
Supplement: Supplementary file 1 — Supplementary Materials: brb371398‐sup‐0001‐SuppMat.docx [file BRB3-16-e71398-s001.docx]

**Reproducibility and Open Science**

To enhance the transparency of this study and facilitate the replication of our findings by the broader research community, all critical code associated with the study has been made publicly available via GitHub.

The open-access code repository can be accessed at:

<https://github.com/huahua-123553/Fusion-of-Multi-Paradigm-EEG-Microstate-Features.> All code is provided under the MIT License, which permits free use, modification, and distribution, subject to the inclusion of the original copyright notice and permission notice. This commitment to open science aims to lower barriers for validating our results, enable extensions of the current work (e.g., applying the multi-paradigm microstate feature fusion approach to other EEG datasets), and foster collaborative advancements in the field of EEG microstate analysis.
